# Cotrimoxazole plasma levels, dialyzer clearance and total removal by extended dialysis in a patient with acute kidney injury: risk of under-dosing using current dosing recommendations

**DOI:** 10.1186/2050-6511-14-19

**Published:** 2013-04-03

**Authors:** Christian Clajus, W Nikolaus Kühn-Velten, Julius J Schmidt, Johan M Lorenzen, Daniel Pietsch, Gernot Beutel, Jan T Kielstein

**Affiliations:** 1 Department of Nephrology and Hypertension, Medical School Hannover, Hannover, Germany; 2 Medical Laboratory Bremen, Bremen, Germany; 3 Institute of Clinical Chemistry, Medical School Hannover, Hannover, Germany; 4 Department of Hematology, Hemostasis, Oncology, and Stem Cell Transplantation, Medical School Hannover, Hannover, Germany

**Keywords:** Antibiotics, Pharmacokinetics, Intensive care unit, Drug monitoring

## Abstract

**Background:**

Dosing of antibiotics in critically ill patients is challenging. It becomes even more difficult if renal or hepatic impairment ensue. Modern means of renal replacement therapy are capable of removing antibiotics to a higher rate than decades ago, leaving clinicians with a high degree of uncertainty concerning the dose of antibiotics in this patient population. Cotrimoxazole, a combination of trimethoprim (TMP) and sulfamethoxazole (SMX) is frequently used in the treatment of several infections including Pneumocystis jirovecii pneumonia (PCP).

**Case presentation:**

Here we describe a patient with acute kidney injury in which we investigated the TMP and SMX levels during the course of an ICU stay. Cotrimoxazole was administered every six hours i.v. in a dose of TMP/SMX 15/75 mg/kg/day. Extended dialysis was performed with a high-flux dialyzer. Blood samples, as well as pre- and postdialyzer samples and aliquots of the collected spent dialysate were collected.

Observed peak concentrations (Cmax) were 7.51 mg/l for TMP and 80.80 mg/l for SMX. Decline of blood levels during extended dialysis (TMP 64%; SMX 84%) was mainly due to removal by the dialysis procedure, illustrated by the high dialyzer clearances (median of 4 extended dialysis sessions: TMP 94.0 / SMX 51.0 ml/min), as well as by the absolute amount of both substances in the collected spent dialysate (median of 6 extended dialysis sessions: TMP 556 mg / SMX 130 mg). Within the limitation of a case report our data from 4 consecutive extended dialysis sessions suggest that this procedure substantially removes both TMP and SMX.

**Conclusions:**

Dose reduction, which is usually advocated in patients with acute kidney injury under renal replacement therapy, might lead to significant under-dosing. Pharmacokinetic studies for TMP/SMX dosing in this patient population are necessary to allow adequate dosing.

## Background

Cotrimoxazole, a fixed 1:5 combination of trimethoprim (TMP) and sulfamethoxazole (SMX), has a broad spectrum of activity against gram-positive and gram-negative bacteria as well as selected protozoa. It is approved for the treatment of acute exacerbation of chronic bronchitis, otitis media (in *S. pneumoniae* sensitive cases only), traveler’s diarrhea, shigellosis, urinary tract infections, as well as for Pneumocystis jirovecii pneumonia (PCP) prophylaxis and treatment. After oral administration the drug is almost completely absorbed. The protein binding for TMP/SMX is 45/68%. Both compounds are excreted in urine as metabolites and as unchanged drug. Consequently the normal half-life of TMP/SMX (6-17/9 hours) is prolonged in renal impairment. For the treatment of PCP a TMP/SMX dose of 15-20/75-100 mg/kg/day TMP/SMX divided in four doses per day for 14–21 days is recommended. In this patient population acute kidney injury is frequently seen [1]. However there is scarce data to guide dosing in patients undergoing renal replacement therapy. For patients on thrice weekly hemodialysis a dose of 5/20 mg/kg (TMP/SMX) 3 times/wk after hemodialysis is recommended [2]. Currently the recommended dose in patients undergoing CVVH (continuous veno-venous hemofiltration) therapy is 5–15 mg/kg/d TMP. A recent case report suggests that this dose is not sufficient when a filtration rate of 29 ml/kg/min is used [3]. Moreover, there are no data on extended dialysis, a hybrid of continuous and intermittent renal replacement therapy, which is increasingly used throughout the world [4,5]. Extended dialysis removes various antibiotics more efficiently compared with standard intermittent hemodialysis three times a week or continuous renal replacement therapy [6-8]. To improve the pharmacokinetic information available to clinicians, as recently asked for by a KDIGO (Kidney Disease: Improving Global Outcomes) working group [9], we here report for the first time data on cotrimoxazole serum levels, dialyzer clearance and dialysate concentrations from a critically ill dialysis patient with PCP undergoing extended dialysis. Written informed consent was obtained from the patients legal representative (wife) for publication of this case report. A copy of the written consent is available for review by the Editor-in-Chief of this journal.

## Case presentation

A 74 year old Caucasian male (186 cm, 84 kg) with a five year history of biopsy proven cANCA (cytoplasmic antineutrophil cytoplasmic antibody) positive vasculitis, leading to stable chronic kidney disease (KDOQI-stage 4), was admitted to the intensive care unit of our tertiary care hospital with tachypnea (respiratory rate of 20/min) and a peripheral oxygen saturation of 88% under 12 l oxygen per nasal cannula. The patient had been on immunosuppressive therapy for 5 years. Initially he received azathioprine and corticosteroids. After two years the immunosuppressive regime was changed to corticosteroids alone due to a carcinoma of the bladder. Two months prior to admission the patient developed a pulmonary relapse (hemoptysis) of his vasculitis and was treated with two bolus infusions of Cytoxan (500 mg/m^2^). Two weeks prior to the scheduled third i.v. bolus of Cytoxan the patient had complained about progressive dyspnea with unproductive cough. His general practitioner prescribed amoxicillin/clavulanic acid in response to elevated inflammatory markers. Aside from his vasculits his past medical history was significant for a myocardial infarction, mitral- and aortic valve insufficiency, arterial hypertension, thrombosis of the femoral vein, secondary hyperparathyreoidism and subacute atherosclerotic encephalopathy. In addition to the tachycardia the physical exam on admission to the intensive care unit was remarkable for a 2/6 systolic murmur. His blood pressure was 128/64 mmHg, heart rate 79 bpm and auricular temperature 36.2°C. Chest X-ray showed a marked interstitial pneumonitis. Material obtained during bronchoscopy confirmed the diagnosis of PCP. Antibiotic therapy with TMP 800 mg/day and SMX 4 g/day [respectively TMP: 10 mg/kg/day and SMX: 48 mg/kg/day] divided in four doses was started. In addition the patient received prednisolone at a dose of 25 mg every 6 hours. Despite this therapy the patient deteriorated under non-invasive patient's ventilation and therefore needed mechanical ventilation. Concomitantly he developed acute on chronic oliguric kidney injury. Extended dialysis was performed (mean daily dialysis dose 74.4 ± 12.6 l), which represents the institutional standard of care. In response to expected additional clearance by the dialysis the antibiotic dosages were adjusted to TMP 1.28 g/day and SMX 8 g/day [respectively TMP: 15 mg/kg/day and SMX: 95 mg/kg/day]. Two days after therapy initiation the patient stabilized and was extubated. A repeated bronchoscopy was negative for pneumocystis jirovecii infection and the dose was reduced to TMP 640 mg/day and SMX 3.2 g/day [respectively TMP: 7 mg/kg/day and SMX: 38 mg/kg/day]. Extended dialysis was continued due to persisting kidney injury. In the further course the respiratory situation worsened due to viral and bacterial superinfection (herpes-simplex; pseudomonas; gram-positive cocci), requiring re-intubation. The patient could not be weaned from mechanical ventilation and continuously required inotropic substances. Subsequently the patient suffered a myocardial reinfarction and died 35 days after admission on our intensive care unit.

For the determination of TMP/SMX levels blood samples were collected after drug infusion, prior to dialysis, under dialysis and post dialysis. Trimethoprim and sulfamethoxazole were quantified in parallel in serum specimens. After protein precipitation with acetonitrile/methanol, matrix components were separated by isocratic reverse-phase HPLC (high-performance liquid chromatography), and specific molecule fragments were detected and quantified after electrospray ionisation in a PESciex API2000 triple-quadrupole mass spectrometer. Dialysates were analogously analyzed by HPLC-MSMS. The lower limit of detection was 0.1 mg/l for both substances, and the interassay CV (coefficient of variation) was 10.3% for TMP and 9.9% for SMX, respectively. EDD (extended daily dialysis) was performed using the 75 l GENIUS® batch dialysis system (Fresenius Medical Care, Bad Homburg, Germany) with a polysulphone high-flux dialyzer (F60S, surface area 1.3 m^2^, Fresenius Medical Care, Bad Homburg, Germany) as previously described [7]. The technical details of the system are explained elsewhere [10]. The average dialysis time during this investigation was 442 ± 101 min, and mean blood and counter current dialysate flow was 170 ± 41 ml/min. Vascular access was achieved by a double lumen catheter inserted in the internal jugular vein. The dialyzer clearance was estimated from concentrations before (C_in_) and directly after (C_out_) the dialysis membrane as CL_dial_ = (Fl_in_ · C_in_ − Fl_out_ · C_out_) / C_in_, where the plasma flow in (Fl_in_) and out (Fl_out_) of the dialyzer was estimated using the blood flow, hematocrit and ultrafiltration rate. In addition, total drug removal was estimated by measuring drug concentration in the spent dialysate, since the GENIUS-system permits easy access to the entire amount of substances that had been removed during a dialysis session. All samples were centrifuged at 1300 g for 10 min at 4°C. Plasma was separated and stored at −80°C until analysis. Data are presented as absolute numbers, percentages, and means with corresponding standard deviations (SD) unless otherwise stated.

**Figure 1:**
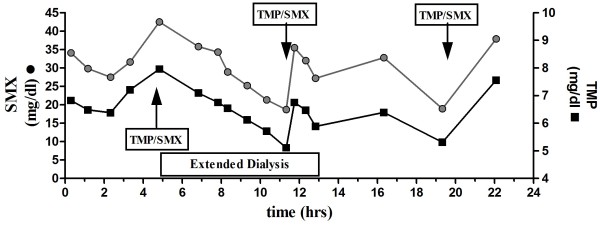
**Time course of plasma Sulfamethoxazole (SMX) and Trimetoprim (TMP) over a time period of 24 hours of an ICU patient with acute on chronic renal failure undergoing extended dialysis.** TMP/SMX were administration indicated by arrows and TMP/SMX. Width of box for extended dialysis corresponds to the time of the procedure.

**Figure 2:**
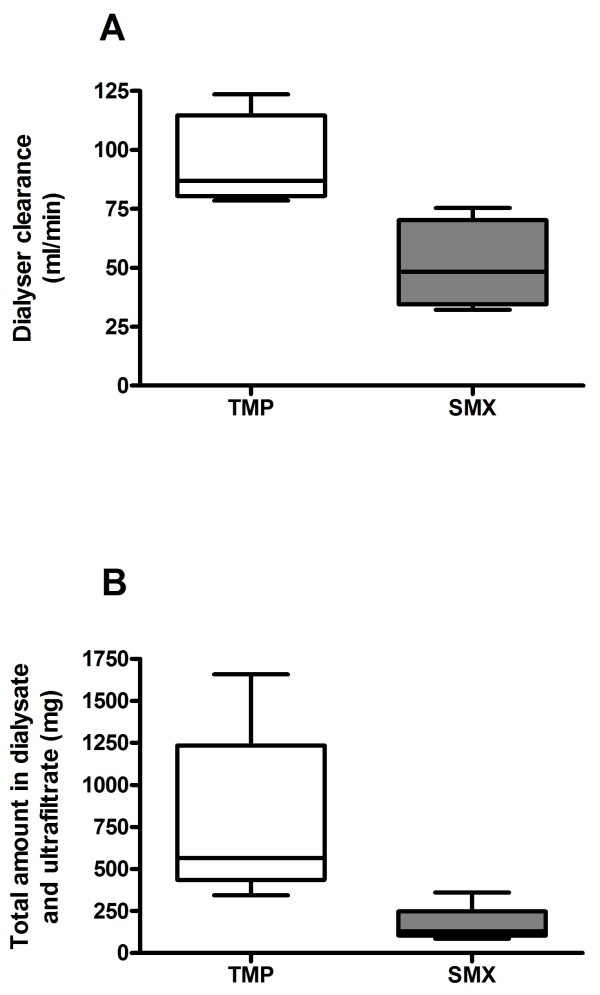
**Box plot of the dialyzer clearance for a polysulphone high-flux dialyzer (F60S, surface area 1.3 m**^**2**^**) for Sulfamethoxazole (SMX) and Trimetoprim (TMP).**

Peak concentrations (immediately after infusion was finished and before RRT was initiated) for TMP was 7.51 ± 1.15 mg/l and for SMX 80.80 ± 3.8 mg/l in a study period of three consecutive days with a dose of TMP/SMX 15/75 mg/kg/day (Figure 1). Concentrations fell to 5.13 ± 1.79 mg/l for TMP (*p* = 0.08) and 38.73 ± 6.61 mg/l for SMX (*p* = 0.007) at the end of dialysis. The dialyzer clearances from 4 consecutive extended dialysis treatments are depicted in Figure 2A. They were 94.0 ± 20.2 ml/min for TMP and 51.0 ± 18.8 ml/min for SMX . Additionally the average total amount of removed drug was 781 mg for TMP and 766 mg for SMX (median TMP 565 (436 - 1234) mg, SMX 130 (104 - 247) mg (respectively 55% for TMP and 55% for SMX of the previously infused dose) per treatment session (Figure 2B).

## Conclusions

Cotrimoxazole is one of the most poorly studied drugs when it comes to dosing under renal replacement therapy. This is especially surprising given the fact that it is used for half a century. The first report on the influence of hemodialysis on TMP/SMX included *3* patients that received twice weekly dialysis for 13 hours using a modified KIIL dialyzer (surface area 1.0 m^2^) indicating that TMP is not removed as is SMX [11]. Back then patients dialyzed twice weekly for 13 hours. Craig and Kunin studied four hemodialysis patients, they did not however provide any data on the coordinates of the dialysis procedure itself, making the interpretation of their data impossible [12]. The Current dosing recommendation in chronic hemodialysis patients is therefore solely based on a 1987 paper by Nissenson et al. [13]. They investigated 16 stable hemodialysis patients that were all dialyzed for 4 h with a 1.0 m^2^ cuprophane dialyzer at a blood flow of 200 ml/min and a dialysate flow of 500 ml/min. Dialysis clearance averaged 38 ml/min for TMP and 42 ml/min for SMX. About 44% of the administered TMP and 57% of the administered SMX were removed during dialysis. Therefore, 50% of the maintenance dose of TMP-SMX should be supplemented after each dialysis session. Recently, Curkovic et al. reporting two patients undergoing CVVHD (continuous veno-venous hemodialysis) showed clearances of TMP (21.5-28.9 ml/min) that were within the range observed in patients with normal renal function (20–80 ml/min). SMX clearance in CVVHDF (continuous veno-venous hemodiafiltration) showed a high variability (18.7, 26.7, and 42.6 ml/min) and exceeded renal clearance values in normal renal function (1–5 ml/min) [3] (Table 1).

**Table 1:** Coordinates of PK parameters and dialysis procedure of our patient undergoing extended dialysis compared to the data from patients undergoing intermittent hemodialysis and CVVH from the literature

**TMP /SMX**	**ED**	**IHD [**[13]**]**	**CVVHDF [**[3]**]**
**n**	**1**	**16**	**2**
Administred dose (mg)	**800/4000**	160/800	160/800
C_max_ (mg/l)	**7.51/80.8**	1.93/41.8	3.73/33.4
Blood flow (ml/min)	**140**	200	150 & 180
Dialyzer clearance (ml/min)	**94/51**	38/42	26/29
Dialyzer surface (m^2^)	**1.3**	1.0	1.8
Dialyzer	**Polysulphone**	Cuprophane	Polysuphone
Treatment time (min)	**442**	240	Not reported

Our patient underwent extended dialysis due to oliguric kidney injury and volume overload. We found peak concentrations of both drugs being in the upper recommended range (TMP 7.51 ± 1.15 mg/l and SMX 80.80 ± 3.8 mg/l) with administration of the standard dose for TMP and SMX for patients with normal renal function [respectively TMP: 15 mg/kg/day and SMX: 95 mg/kg/day]. After dialysis session the concentrations declined rashly. These results imply a significant drug elimination compared with the above described dialysis modality. This hypothesis is confirmed by the calculated dialyzer clearances (TMP 94.0 ± 20.2 ml/min and SMX 51.0 ± 18.8 ml/min; Figure 2). These results exceed by far the renal clearance under physiological conditions. In a work from Paap clearances varied from 1.13 – 103.6 ml/min for TMP and 0.4 – 18.29 ml/min for active SMX in patients with normal renal function [14]. Some explanation may be given when comparing the actual dialysis modalities to the ones used 30 years ago. The blood flow is comparable and the counter current dialysate flow is even less in this study, but the dialyzer membrane has changed to a high-flux-dialyzer with a significant larger surface area. Furthermore, the effective dialysis time (442 ± 101 min) combined with a daily therapy could explain the highly efficient elimination of TMP and SMX.

In conclusion the high elimination rate of TMP and SMX in our patient undergoing extended daily dialysis suggests that maybe old dosing regimens from the time of less efficient renal replacement therapies might not yield adequate dosing. This potential problem is aggravated by the fact that drug monitoring for TMP/SMX is not recommended and determination of a successful therapy is mainly based on clinical parameters [15]. Further pharmacokinetic studies should be performed to develop dosing recommendations for TMP/SMX that keep up with the efficacy of modern renal replacement therapies.

## Consent

Written informed consent was obtained from the patient’s legal representative for publication of this Case report and any accompanying images. A copy of the written consent is available for review by the Series Editor of this journal.

## Competing interests

The authors declare that they have no competing interests.

## Authors’ contributions

NKV conducted the measurement of TMP/SMX. CC, GB, JL and JTK were the treating physicians of the patient reported. CC, JTK and DP evaluated the test results. All of the authors have participated in the discussion and in writing of the submitted manuscript. All authors read and approved the final manuscript.

## Pre-publication history

The pre-publication history for this paper can be accessed here:

http://www.biomedcentral.com/2050-6511/14/19/prepub
